# Gut Microbiota and Behavioural Issues in Production, Performance, and Companion Animals: A Systematic Review

**DOI:** 10.3390/ani13091458

**Published:** 2023-04-25

**Authors:** Bonnie Homer, Jackson Judd, Manijeh Mohammadi Dehcheshmeh, Esmaeil Ebrahimie, Darren J. Trott

**Affiliations:** 1 School of Animal and Veterinary Sciences, The University of Adelaide, Adelaide, SA 5371, Australia; 2 Genomics Research Platform, School of Life Sciences, College of Science, Health and Engineering, La Trobe University, Melbourne, VIC 3086, Australia; 3 School of BioSciences, The University of Melbourne, Melbourne, VIC 3010, Australia

**Keywords:** animals, behaviour, microbiome

## Abstract

**Simple Summary:**

The body is home to a complex, finely balanced ecological community containing commensal, symbiotic, and pathogenic microorganisms that are collectively termed the microbiota. This system of living organisms plays a crucial role in integrating the neural, endocrine, and immune systems via a bidirectional communication pathway known as the gut-brain-immune axis. In concert with these pathways, the microbiota can influence their hosts’ neurological system to have a profound influence on mental health, mood, and behaviour. Despite the abundance of research in human medicine, there is limited literature exploring the connection between abnormal behavioural phenotypes and disturbances in the gut microbiota of animals. This review provides the first compilation of the current progress of gut microbiota analysis in production, performance, and companion animal studies. We assessed the similarities of gut microbiota between animal categories and compared them to the gut microbiota population shifts already associated with human mental health disorders.

**Abstract:**

The literature has identified poor nutrition as the leading factor in the manifestation of many behavioural issues in animals, including aggression, hyperalertness, and stereotypies. Literature focused on all species of interest consistently reported that although there were no significant differences in the richness of specific bacterial taxa in the microbiota of individual subjects with abnormal behaviour (termed alpha diversity), there was variability in species diversity between these subjects compared to controls (termed beta diversity). As seen in humans with mental disorders, animals exhibiting abnormal behaviour often have an enrichment of pro-inflammatory and lactic acid-producing bacteria and a reduction in butyrate-producing bacteria. It is evident from the literature that an association exists between gut microbiota diversity (and by extension, the concurrent production of microbial metabolites) and abnormal behavioural phenotypes across various species, including pigs, dogs, and horses. Similar microbiota population changes are also evident in human mental health patients. However, there are insufficient data to identify this association as a cause or effect. This review provides testable hypotheses for future research to establish causal relationships between gut microbiota and behavioural issues in animals, offering promising potential for the development of novel therapeutic and/or preventative interventions aimed at restoring a healthy gut-brain-immune axis to mitigate behavioural issues and, in turn, improve health, performance, and production in animals.

## 1. Introduction

Gut microbiota research and its relationship to mental health is an emerging research field [1,2,3]. While aspects have been studied relatively extensively in the human field, including laboratory rodent models, other animal-based gut microbiota research and its putative links to adverse behaviour are very much in their infancy [4]. In healthy humans, the gut microbiota is established towards the end of the gestation period and through the process of parturition. A large variety of microbes is acquired from the maternal intestinal microbiota and influenced by the dam’s diet and method of delivery [5,6,7,8]. Maternal microbiota contributes to microbiome diversity throughout the growth stages of the animal, but parturition remains a crucial time for development of the gut microbiota. Autochthonous bacteria present in the gut act in a symbiotic relationship with the host. Both will provide each other with nutrients to sustain life, enhancing protective systems for the host, while the host will provide the bacteria with an ideal environment supporting continued reproduction. Gut bacteria also play an important role in the development of specific aspects of the brain, affecting the growth and maturation of precise regions of neurological white matter [9]. Whilst current and recent multidisciplinary research breakthroughs have significantly improved our understanding of the gut-brain-immune axis in humans, this issue has yet to be adequately addressed in animals other than laboratory rodent models.

Many lines of evidence support the need for a holistic approach to medicine, with detrimental effects in the gut microbiome now recognised as having a much larger role in immunological tolerance, cognitive function, and physiological development of the individual than previously thought. In addition to bacterial abundance, microbiome diversity is an important feature [10]. Human-based research has identified correlations between the abundance of specific bacteria taxa and mental disorders such as bipolar disorder, anxiety, depression, and schizophrenia [11]. While these disorders are not currently recognised or indeed investigated in animals because of the difficulty in establishing and distinguishing specific pathognomonic features relevant to production companion and performance animal health, the findings from human research may play a large role in how veterinarians diagnose and treat behavioural disorders in the future. Although some aspects of the complex relationship between healthy gut microbiota, immune response, and mental health and welfare have been recognised in animals, the current scope of literature is limited; for example, the effects of the large differences in digestive system physiology between fore and hindgut fermenters compared to monogastrics on microbiome profile are largely unknown, limiting established collations of intra- and inter-species similarities and distinctions [11,12,13].

In pigs, many gut bacteria are involved in the breakdown of dietary carbohydrates (which are not processable by the host) into short-chain fatty acids (SCFAs), including acetate, butyrate, and propionate [13]. Additionally, adverse events resulting from sudden shifts in diet followed by microbial community shifts in lactic acidosis are well documented [14]. In humans, many gut bacteria have been shown to influence the proliferation of oligodendrocyte progenitor cells (OPCs), which play a crucial role in the myelination of axons and the development of axon pathways [9]. They are also involved in the physiological development of white matter in the brain, specifically in areas related to cognition and emotional capacity [9]. When gut health is maintained, the host can utilise SCFAs for nutritional purposes and undergo healthy brain development, with each SCFA playing an important role in immune system development, regulation, and neurological function [9,13]. Some bacteria can also produce tryptophan metabolites, enabling the host to utilise gut-derived neurotransmitter precursors such as indole. Some species of bacteria can even directly produce neurotransmitters such as serotonin and melatonin, which are crucial for normal brain function and sleep regulation [13,15,16,17]. These metabolites play an important role in mental health and behavioural changes.

This review will provide a comprehensive analysis of the current state of animal-based gut-immune-brain axis research. It will compare three categories of animals with one example from each: production animals (pigs), performance animals (horses), and companion animals (dogs).

## 2. Methods

This systematic review adheres to the Preferred Reporting Items for Systematic Review and Meta-Analyses (PRISMA) framework [18]. Eligible articles had to meet the following inclusion criteria: (1) the study must be relevant to the research topic; (2) it must be published in English and peer-reviewed with the full text available; and (3) it must have been published within the last 10 years, unless no recent study was available.

A systematic search was conducted in PubMed for eligible articles from 1 August to 30 September 2022 using the search strategy (microbiota OR microbiome) AND (gut OR gastrointestinal OR “gut-brain”) AND (behaviour OR behavioural OR stereotypies) for each animal species of interest: porcine (pigs OR swine OR porcine), equine (horse OR equine), and canine (dog OR canine). Further studies were identified by reviewing the bibliographies of inclusion articles. Additionally, a separate search was conducted to review the most recent human literature with the specific aim of identifying which bacterial taxa are associated with human mental health and behavioural disorders and their putative role in disease pathogenesis. Eligible articles met the same inclusion criteria, with the exception that the search was refined to only include meta-analysis and systematic review articles published since 2020. The search strategy included (microbiome OR microbiota OR bacteria) AND (gut OR gastrointestinal) AND (“mental disorder”), with the additional key search terms “short chain fatty acids”, “neurotransmitters”, “HPA axis”, and “immune pathway”.

A total of 372 articles were identified from primary database searches using the above-mentioned search terms. Following an initial screening of the abstracts of the 372 articles, 81 were deemed relevant and were selected for a full-text analysis (291 were excluded). Of these articles, 61 did not meet the outlined inclusion criteria (investigating the gut microbiota’s association with behaviour/brain function and development/gut-brain-immune axis) and consequently were excluded. The remaining 20 articles were included in our systematic review, as were an additional 19 that were identified from the bibliographies of relevant articles. In total, 39 studies met the outlined inclusion criteria and were selected for this systematic review, of which 5 investigated gut microbiota composition in pigs, 11 in horses, 9 in dogs, and 14 in human patients with mental disorders. 

## 3. Results

Gut microbiota describes the collection of bacteria, archaea, and eukarya that have colonised the gastrointestinal tract [19,20]. The gut microbiota has a vital role in integrating the neural, endocrine, and immune systems via a bidirectional communication pathway termed the gut-brain-immune axis. The brain can influence gut motility, secretion, and permeability, which in turn affects the gut microbiota. Conversely, the microbiota can produce various metabolites, which act via the vagus nerve, the blood-brain barrier (BBB), and through modulation of the hypothalamic-pituitary-adrenal (HPA) axis and the immune system [11,21,22,23,24]. Some of the important microbial products include SCFAs, neurotransmitters, and their precursors. These products have neuroactive properties that can influence their hosts’ central nervous systems (CNS) and impact emotion, mood, and behaviour [11,23,25,26].

Some commensal anaerobic microbes, including genera within the Bacteroidetes and Firmicutes phyla, ferment indigestible polysaccharides to produce SCFAs—butyrate, propionate, and acetate [23]. These compounds can act locally as the major energy source for intestinal cells. They are also involved in maintaining the intestinal barrier, as they preserve tight cellular junctions and enhance the production of mucus and antimicrobial peptides [23,27]. Alternatively, SCFAs can have systemic effects, which can be achieved indirectly via the stimulation of the enteroendocrine cells (EECs) to produce hormones that either enter systemic circulation or modulate the vagus nerve. SCFAs can also directly enter the circulation and cross the blood-brain barrier (BBB), influencing the CNS. In the CNS, SCFAs influence BBB permeability by upregulating the expression of tight-junction proteins, limit neuroinflammation by decreasing the activity of microglia, stimulate neurogenesis, and modulate neural homeostasis and function [22,23,28,29,30]. Additionally, SCFAs can limit both the immune and endocrine responses as they have anti-inflammatory properties, activate T-regulatory cells, and decrease the expression of proteins involved in the HPA axis [23]. Collectively, these interactions can influence cognition, emotion, and behaviour [31,32,33].

Neurotransmitters are widely known to influence emotions and behaviour [22,34,35]. Dopamine is associated with feelings of pleasure, motivation, and reward. Serotonin is colloquially known as the “happiness hormone” and is involved in the regulation of mood along with sleep and digestion. Acetylcholine and glutamate are involved in learning and memory. Norepinephrine is linked to alertness, fear, anger, and stress, while gamma-aminobutyric acid (GABA) is known to have a calming effect, among others [22,34,35]. 

These neurochemicals (and their precursors) are synthesized by neurons as well as specific gut microbes [23,25]. The bacterial genera currently known to be responsible for producing neurotransmitters and/or their precursors include the following [26,36]:

- GABA produced by *Lactobacillus* and *Bifidobacterium*;
- Norepinephrine produced by *Escherichia*, *Bacillus*, and *Saccharomyces*;
- Dopamine produced by *Bacillus*;
- Acetylcholine produced by *Lactobacillus*;
- Serotonin produced by *Escherichia*, *Enterococcus*, *Candida*, and *Streptococcus*;
- Tryptophan produced by *Clostridium*, *Bacteroides*, *Escherichia*, *Burkholderia*, *Streptomyces*, *Pseudomonas*, and *Bacillus*.

These compounds can act locally on EECs to affect gastrointestinal function and regulate the enteric immune response [37,38,39,40,41,42]. Alternatively, they can influence the CNS [40,43,44]. Although gut-derived neurotransmitters themselves are unable to cross the BBB, they act as signalling molecules on specialised EECs—neuropod cells—that synapse with the vagus nerve to transmit signals to the brain [11]. Conversely, microbes can also produce neurotransmitter precursors such as phenylalanine, tyrosine, and tryptophan, which can cross the BBB and become synthesised into functional neurotransmitters [22]. Furthermore, many common gut microbiota inhabitants, such as *Bacteroides* and Enterobacteriales, have been shown to metabolise tryptophan into indole derivatives and SCFAs. Tryptophan metabolites can pass through the BBB, with indole decreasing pro-inflammatory responses from astrocytes and SCFAs playing a role in the regulation of microglial homeostasis. This has been demonstrated in a mouse model in which 11C- and 13C-labelled acetate entered systemic circulation before crossing the BBB into the hypothalamus, where it was integrated into pathways including the glutamate–glutamine and GABA cycles [45]. Within the CNS, these neurotransmitters impact cognition and behaviour by influencing learning, emotions, and impulse control [46]. 

These metabolites remain at equilibrium in a healthy individual as the gut-brain-immune axis maintains a homeostatic balance of gastrointestinal bacteria. Diet changes, antibiotic use, stress, or a pathogenic infection can disrupt homeostasis, and dysbiosis results. This can involve a reduction in bacterial diversity, a loss of commensal bacteria, and/or an overgrowth of pathogenic bacteria [47]. As the gut microbiota are responsible for producing significant amounts of neurotransmitters (and precursors) as well as SCFAs in the body, changes in bacterial composition can impact the concentrations of these neuroactive chemical messengers and, in turn, the emotions and behaviours they influence.

As a consequence of altered SCFA concentrations, dysbiosis can lead to ‘leaky gut’: a syndrome involving intestinal inflammation, impaired permeability, and subsequent translocation of microbial metabolites, antigens, and enteric corticosterone into systemic circulation [21,22,24,26]. These molecules can cross the BBB or modulate the vagus nerve to activate the HPA axis, or they can stimulate systemic inflammation, with the subsequent release of cytokines and prostaglandins causing inflammatory activation [21]. This results in a stress response, in which physiological changes can affect behaviour, including cognitive behaviour, fear, hyperresponsiveness, and aggression [48]. Additionally, the activated HPA axis can influence gut motility, secretions, and permeability—thus changing the gastrointestinal environment and exacerbating dysbiosis. The release of catecholamines from the HPA axis can also directly stimulate the growth of pathogenic bacteria [21,49]. Luo et al. (2018) demonstrated the importance of gut microbiota on HPA axis response regulation in germ-free mice [50]. These models displayed a hyperresponsive HPA axis reaction to stressors, with alterations evident in blood markers as well as an upregulation of the genes in the hippocampus encoding proteins involved in the HPA axis response. Proper functioning of such a hyperactive HPA axis can be restored following administration of probiotics containing *Lactobacillus* and *Bifidobacterium* [21].

Recent research has also explored the effects of dysbiosis during early life development. As establishment of the microbiome coincides with the development of the immune and nervous systems, dysbiosis caused by some antibiotic treatments during this critical developmental period has been shown to affect both systems and, in turn, influence the development of cognitive and behavioural disorders [25]. A rodent-based study involving dysbiosis during microbiota colonisation (initiated by antibiotic treatment during pregnancy) demonstrated reduced exploratory behaviour effects on offspring for the entire duration of the study [24]. Dysbiosis, initiated by maternal immune activation, poor diet, infection, or antibiotic use, can cause systemic inflammation and initiate a dysregulated HPA axis and a hyper-reactive immune response [21,25]. Additionally, dysbiosis may result in the underdevelopment of the immune system with fewer and smaller lymph nodes [24]. 

Abnormal neuronal development and associated cognitive and neurobehavioural disorders may also occur because of dysbiosis and microbial-derived neuroactive metabolites [51]. The prefrontal cortex is particularly at risk of developmental problems associated with dysbiosis as it undergoes myelination at a later, more vulnerable period [25]. Disturbances in the myelination of the prefrontal cortex have recently been linked to the development of poor social behaviour in humans [25]. Additionally, increased BBB permeability associated with early life dysbiosis can further contribute to the development of a hyperresponsive immune system as more inflammatory mediators, such as cytokines, can cross the BBB [24]. An increased level of cytokine expression in the brain, particularly in the hippocampus, has been observed in mice exposed to prenatal stress and dysbiosis [52]. In humans, dysbiosis has also been linked to increased activation of microglia and astrocytes, which influence synaptic development and neuroplasticity [24,26]. Furthermore, neurological disturbances associated with the disruption of gut microbiota during initial bacterial colonisation cannot be reversed [24,26]. It may result in the development of permanent changes in behavioural phenotype if it occurs during the time window crucial for microbiota colonisation and neural and immune development [24,26]. Altogether, dysbiosis can be involved in temporal behavioural changes if it occurs in more mature, developed animals, or it may result in the development of permanent changes to behavioural phenotypes if it occurs during the window of crucial colonisation and neural and immune development.

### 3.1. Humans

Because of the microbiota’s profound influence on emotion, mood, and behaviour, human medicine has begun to intricately examine the gut microbiota to identify specific bacterial taxa that are commonly associated with mental disorders, such as depression (D), anxiety (A), bipolar disorder (BD), and schizophrenia (SZ) [26,27,53,54,55,56,57]. The selected search terms identified 14 research papers specifically exploring the association between mental health disorders and alterations in microbiota populations. Amongst these studies were numerous recent systematic literature reviews and meta-analyses (2020–2022) that amalgamated the correlations identified between population shifts in specific gut microbe taxa and mental health disorders. Studies documenting mental health disorders and their association with increased or decreased relative abundance of different microbial populations are shown in Table 1. Studies in Table 1 are performed on both male and female individuals, mainly by analysis of faecal samples. 

**Figure 1:**
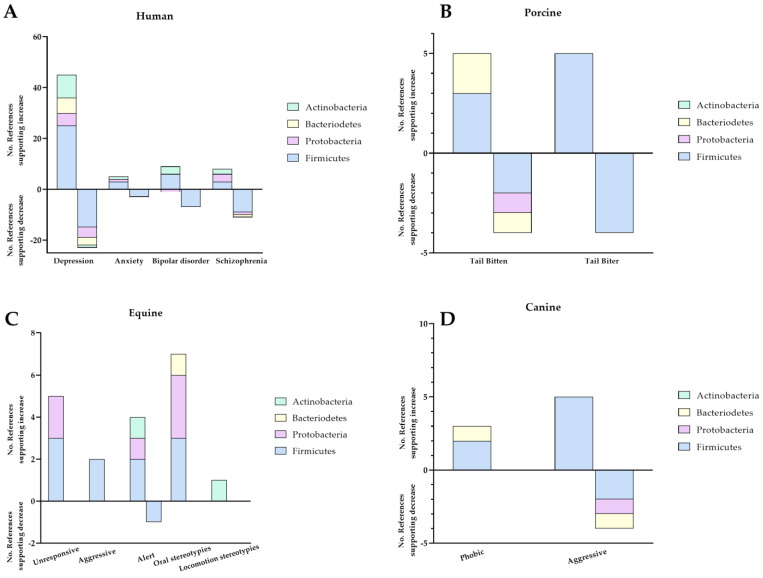
Number of references reporting increase or decrease in the levels of Firmicutes, Protobacteria, Bacteriodetes, and Actinobacteria with depression, anxiety, bipolar disorder, schizophrenia, tail bitten, tail bitter, unresponsive, aggressive, alert, oral stereotypies, locomotion stereotypies, phobic, and aggressive in four selected species: human (**A**), porcine (**B**), equine (**C**), and canine (**D**).

Figure 1, designed based on the data in Table 1, displays a shift in microbiota composition in humans across four major phyla of gut microbes (Actinobacteria, Bacteriodetes, Protobacteria, and Firmicutes) for depression, anxiety, bipolar disorder, and schizophrenia.

Some of the reviewed articles also analysed microbiota diversity using alpha and beta diversity indexes. Alpha diversity measures the richness of microbial taxa in individual microbiome samples, whereas beta diversity assesses the variability in species diversity between multiple samples [58]. The articles consistently identified that although there are no significant differences in alpha diversity between the investigated patients and controls, there are differences in beta diversity [26,27]. The gut microbiomes of patients with mental health disorders consistently display an enrichment of pro-inflammatory and lactic acid-producing bacteria, a reduction in butyrate-producing bacteria, and an abundance of bacteria associated with glutamate and GABA metabolism [25,26,27,53,54,55,56,57].

Lactic acid-producing bacteria can have beneficial impacts when present in the gut at moderate levels. This includes the regulation of metabolism, protection against pathogenic bacteria, lactate production, and immunomodulatory effects [27]. As a result, *Lactobacillus* is commonly included as a probiotic in many human health foods and has even been associated with decreases in stress and anxiety behaviours [6,25,26,59]. However, if lactic acid-producing bacteria become overabundant, lactic acid accumulation in the gut occurs and can have detrimental impacts. Higher levels of gut *Lactobacillus* are seen in A, D, SZ, and BD; increases in *Streptococcus* in the gut have been identified in BP and D; and higher relative abundances of *Enterococcus* are unique to D; *Escherichia/Shigella* in SZ; and *Bifidobacterium* in BD [25,26,27,53,54,55,56,57].

Butyrate-producing bacteria are crucial in maintaining gut barrier integrity. Reduced butyrate production can result in impaired intestinal permeability and, in turn, the translocation of bacteria and their products into the systemic circulation and subsequent systemic inflammation [25]. Reduced abundance levels of *Coprococcus* and *Faecalibacterium* in the gut are seen in A, D, SZ, and BD, whereas lowered *Roseburia* is unique to BD and SZ [25,26,27,53,55,56,57]. Interestingly, administration of butyrate has reversed behavioural hyperactivity and depressive- and manic-like behaviours in experimental settings with mice and pigs [23,60]. 

Alterations in the levels of bacteria involved in glutamate metabolism are also common in patients with mental disorders. Increases in *Lactobacillus* are evident in A, D, SZ, and BD, whilst elevated levels of *Alistipes*, *Oscillbacter*, *Eggerthella*, and *Parabacteroides* are unique to D; *Bifidobacterium* to BD; and *Enterococcus* to D and BD [25,26,27,53,55,56,57]. Some studies propose that these microbes increase the utilisation and depletion of glutamate and subsequently increase the synthesis of GABA, an inhibitory neurotransmitter associated with anxiety and depression-related behaviours [6,61].

### 3.2. Animals

In animals, dysfunctional, repetitive behaviours are often referred to as “stereotypies”, and they include tail and bar biting and pacing in pigs; crib-biting and pacing in horses; and tail chasing, fly snapping, and light/shadow chasing in dogs [62]. These behaviours have traditionally been considered to be expressed as a response to a suboptimal environment and boredom [62]. However, research in various species has identified poor nutrition as another leading factor in their manifestation, which likely influences the microbiome [62,63]. Depending upon the hosts’ nutrition and the food source provided to the gut microbes, different populations of gut microbes will prosper, and, in turn, their metabolites will differ in abundance [64,65]. As discussed, some of these metabolites can impact the host’s CNS via a myriad of pathways, which in turn may affect behaviour. Several recent studies (2019–2022) investigated correlations between population shifts in specific gut microbe taxa and behavioural issues in porcine, equine, and canine hosts. Studies documenting behavioural conditions in animals and their association with increased or decreased relative abundance of different microbial populations are shown in Table 2. 

The frequencies of reported population shifts across four major phyla of gut microbes (Actinobacteria, Bacteriodetes, Protobacteria, and Firmicutes) exhibiting abnormal behavioural phenotypes are displayed in Figure 1.

### 3.3. Production Animals—Pigs

The selected search terms identified three papers linked with behavioural problems in pigs [4,66,73]. Tail biting is a widespread and serious issue in intensive pig farms. A recent study hypothesised that tail-biting in pigs reduced the net profit by up to USD 23.00 per pig, causing millions of dollars of loss annually to the pork industry [15]. Regarding tail biting and the porcine microbiome, no change in alpha diversity was found between animals displaying tail-biting and the control group [66,73]. However, a difference in beta diversity between the tail-biters, the victims, and the control groups was a consistent feature [66,73]. Defined changes were difficult to correlate between studies because of the high level of variability in the gut microbiota throughout the life of a pig. There was a relative increase in abundance of some families and orders of Firmicutes (specifically Clostridiales [*Ruminococcocus*, *Lachnospiriceae,* and Clostridiales Family XII]) and a relative decrease in the abundance of other Firmicutes (specifically *Lactobacillus* spp.) in pigs displaying tail-biting behaviour, pigs that were victims of tail-biting, and pigs displaying other anxiety behaviours [66,73].

Lactobacilli possess the ability to modulate the expression of the host’s immune pathways, creating greater cross-talk between host immune cells and the other autochthonous gut bacteria [74,75,76]. They are recognised as antagonists to pathogenic bacteria such as *E. coli* and *Clostridia*, as well as decreasing the production of stress-induced cortisone and modulating GABAergic and serotonergic signalling pathways in multiple regions of the brain [5,6]. There is evidence that the supplementation of *Lactobacillus* strains alters the anxiety state in piglets, lowering anxiety levels and altering responsive behaviour when presented with a potential threat [73]. 

Within the Firmicutes, an increase in the relative abundances of Family XIII AD3011 and *Ruminiclostridium* was observed in pigs performing tail-biting, compared to a relative increase in *Butyrivibrio* and *Alloprevotella* in victims. Victims of tail-biting also showed a decrease in the relative abundance of *Prevotella* 7 and *Ralstonia* genera when compared to the control groups [4]. However, the apparent decrease in *Prevotella* abundance in victims was contradicted in other porcine studies, as well as human and rodent models of anxiety and depression, which instead show concomitant increases in the relative abundance of *Prevotella* in stressed animals [4].

A relative increase in *Prevotella/*Prevotellaceae, *Coprococcus*, and *Eubacterium coprostanoligenes* is reported in pigs displaying fearful and fear-associated behaviours in response to a novel object test as compared to pigs displaying unstressed behaviours [4]. Population levels of bacteria such as *Coprococcus* are known to coincide with genes associated with dopamine biosynthesis in the intestinal microbiota, meaning they have an inverse relationship with stress and are generally associated with a higher quality of life [4]. A reduction in their population is commonly associated with mental disorders such as Parkinson’s disease [77]. The mentioned inverse relationship between *Coprococcus* and stress in humans contradicts the apparent increase in *Coprococcus* in piglets showing increased signs of anxiety. Abundant *Prevotella* populations, however, are recognised to be positively associated with depression and anxiety in both rodents and humans, which directly correlates with the increased levels of these microbes in pigs failing the stress test [4]. As many of these bacterial taxa are linked both positively and negatively with dysbiosis associated with human mental-health disorders, as well as other animal behavioural issues, further studies are required to shed more light on these questions.

### 3.4. Companion Animals—Dogs

The selected search terms identified seven research papers exploring the associations between behaviour and the gut microbiome of companion animals, specifically canines [46,47,61,71,72,78,79]. All articles were in agreement that the microbiota of normally behaving dogs includes abundant Firmicutes (including the Clostridiales: *Ruminococcus, Faecalibacterium*, and *Dorea*) and Bacteroidetes, whereas Actinobacteria, Fusobacteria, and Proteobacteria are minor components [47,61,71,72]. The relative abundance of bacteria significantly differed in dogs displaying behavioural problems such as aggression, separation anxiety, and phobia [46,72,78]. Firmicutes consistently showed a relative abundance increase across all abnormal behavioural phenotypes [61,71,72]. The microbiomes of phobic dogs were characterised by an enrichment of *Lactobacillus*. This genus of bacteria is known to produce GABA and its precursor, glutamine. It has been reported that in fearful dogs, the increased glutamine plasma concentration is associated with a higher abundance of *Lactobacillus* in the faecal microbiome [78]. Overall, the microbiota of phobic dogs displayed changes in beta diversity and had low biodiversity compared to the microbiota of both normal and aggressive dogs [61,72]. In all studies, aggressive dogs were characterised by a relative elevation in Firmicutes and a reduction of *Bacteroides* [61,71,72]. *Bacteroides* are known to be key tryptophan producers, and so, when dysbiosis results in their reduction, tryptophan is likely to decrease along with its derivative, serotonin. Interestingly, aggressive behaviour has been linked to low serotonin levels in humans [26,61,80] and dogs [81]. Administration of probiotics containing *Bacteroides*, specifically *B. fragilis*, has been shown to reduce anxiety-related behaviours, such as aggression, in laboratory animal studies [82]. The use of probiotics, specifically *Bifidobacterium longum* (BL999), in dogs to modify stereotypical behaviours has been investigated [79]. This treatment resulted in a significant improvement in anxious behaviour with reductions in barking, jumping, spinning, and pacing, as well as increased exploratory behaviour [79].

### 3.5. Performance Animals—Horses

The selected search terms generated 11 research papers specifically exploring the equine gut microbiome and behaviour. Upon analysis, all articles identified poor nutrition as the leading factor in the development of equine behavioural problems, such as alert and hyper-reactive behaviour, aggression (e.g., kicking and biting), and repetitive stereotypies (e.g., crib biting and weaving) [49,70,83,84,85,86,87]. This is likely because modern-day domesticated horse management has deviated significantly from a horse’s natural way of life. Traditionally, equines would slowly and continuously graze on high-fibre pasture throughout the day, whereas nowadays they receive large, high-starch rations during set “mealtimes” [87]. These high-starch diets induce changes in hindgut microbiota, with decreases in Ruminococcaceae and increases in *Streptococcus* evident. These equines also exhibit abnormal stereotypical behaviours and hyper-reactivity at a higher frequency than those on high-fibre rations [49,69,70,85,87,88]. Across all abnormal behavioural phenotypes analysed in equines, the microbiota also showed increases in Firmicutes, exclusively the order Clostridiales. Some increases were also observed in Proteobacteria [49,69,70]. Mach et al. (2021) recently provided a quantitative analysis by using microbiability (m2) to assess the cumulative effects of the gut microbiota on behavioural phenotypes in 185 horses [49]. Overall, abnormal behavioural traits had m2 >15%, indicating a strong relationship between specific behaviours and overall microbiota composition. Specifically, oral stereotypies had the highest association at 24.2%, followed by locomotion stereotypies at 16.2%, aggressiveness at 13%, and hypervigilance at 9%. Thus, the initial studies provide promising indications that unfavourable behavioural phenotypes could potentially be improved by modulating the microbiome. Interestingly, Johnson et al. (1998) discovered that the incidence of these behaviours was reduced when horses were supplemented with virginiamycin—a narrow-spectrum antibiotic within the streptogramin family known to inhibit the growth of Gram-positive, lactic acid producers in the gut, including *Streptococcus* [85], which is also potentially associated with gut carbohydrate overload-induced laminitis [89]. 

### 3.6. Cross-Species Comparisons

Population shifts of Firmicutes, particularly those of the families Clostridiaceae, Lachinospiraceae, Oscillospiraceae, and Lactibacilaceae, displayed the strongest association with behavioural and mental disorders across all animal species and humans. These bacteria are responsible for producing many of the SCFAs, and therefore, a shift in the abundances of Clostridiaceae, Lachinospiraceae, Oscillospiraceae, and Lactibacilaceae can result in changes in SCFA concentrations, which may in turn increase gut permeability [90]. Of particular significance are *Streptococcus*, *Lactobacillus*, and species of the family Lactibacillaceae, which showed an overwhelming increase in abundance in humans and dogs displaying a range of abnormal behavioural phenotypes. Pigs were the exception to that rule, as tail biters had decreased levels of *Lactobacillus* spp. All species showed changes in the family Oscillospiraceae, with relative increases seen in horses displaying stereotypies, pigs displaying tail biting, and humans with depression; a decrease was observed in aggressive dogs. All species analysed in this study also showed abundance changes in the family Lachinospiraceae. 

Within the phyla of Actinobacteria and Protobacteria, humans exclusively displayed abundance changes in Enterobacteriales, Desulfovibrionales, Pasteurellales, Bifidobacteriales, and Coriobacteriales, whilst horses showed unique changes across other orders. There was, however, some consistency in the relative decrease in abundance of the order Burkholderiales (phylum Protobacteria) in depression across some species, including humans, tail-bitten pigs, and aggressive dogs. Meanwhile, aggressive horses and tail-biting pigs both displayed a relative increase in the abundance of *Anaeroplasma*. 

Horses expressing hyperresponsive and stereotypical behaviours shared similar microbiota changes as schizophrenic patients, with an identified abundance increase of Succinivibrionaceae in horses and *Succinivibrio* spp. in schizophrenia [51,68,85,88]. Additionally, both displayed a reduction in Ruminococcaceae [51,68,85,88]. Similarities in microbiota changes also exist between humans with depression and animals expressing depressive-like characteristics. As an example, the gut microbiota of tail-bitten pigs in depressive-like behaviour showed the following changes: (1) exclusive changes in the abundance of the phylum Firmicutes; (2) a relative increase in the abundance of *Blautia* spp.; (3) a relative decrease in the abundance of *Prevotella*; and (4) a relative increase in the abundance of *Alistipes* species [66,73]. Relative increase in the family of Rikenellaceae is also reported in phobic dogs [72]. 

An increase in the relative abundance of Erysipelotrichaceae has been observed in humans with depression [91,92,93] and horses expressing withdrawn and unresponsive behaviours. Phobic dogs also shared similar relative increases in *Lactobacillus* as seen in humans with depression and anxiety. All these data demonstrate that an association exists between abnormal behavioural phenotypes and microbiota population shifts, not only within individual species but also across species. This strongly indicates the need for future research exploring the effects of modifications to the gut microbiota as a means to mitigate adverse behaviours in animals. 

## 4. Discussion

Despite there now being a considerable number of published papers on associations between gut microbiota and brain function in production, companion, and performance animals, we are only at the beginning of our mechanistic understanding of these complex interactions. Many factors alter the diversity and composition of gut bacteria in each host species and can influence the observed association between gut microbiota and behaviour. Furthermore, the majority of published microbiome studies have been carried out by partial 16S rRNA gene sequencing. Partial 16S rRNA gene sequencing has lower power in microbiome profiling compared to the newer technologies of full-length 16S rRNA sequencing [94,95]. There are specific stages of life in which an animal’s gut microbiota is likely to be strongly influenced by external factors, including diet, a variety of stressors, nutrient deficiencies, antimicrobial treatments, or spontaneous events known to cause dysbiosis [64,96]. 

In production, performance, and companion animals, methods for preventing dysbiosis can include strict attention to nutritional requirements, including nutraceuticals; the use of feed additives such as SCFAs, acidifiers, prebiotics, and probiotics; and the reduction of stressful events. Feed additives, for example, have been shown to be extremely beneficial in the maintenance of gut health in specific animals. Oral administration of butyrate to pigs can affect glucose metabolism in some regions of the brain, including the ventral hippocampus, which is known to be related to stress and emotions [60]. While these types of studies are in their early stages, future multidisciplinary studies could include careful measurement of behavioural changes using ethograms to determine the impact of feed additives on stress responses, aggressive behaviour, and fearfulness. Inclusion of specific spices in the diet, such as *Curcuma longa* L., *Piper nigrum* L., *Capsicum anuum* L., and *Zingiber officinale* L., can increase serotonin 5-HT1A receptors in the hippocampus and enhance serotoninergic system regulation and neurotrophic factor expression in the hippocampus and prefrontal cortex [97,98]. The hippocampus and prefrontal cortex are the main areas of the brain affected by most mood disorders such as anxiety [98,99]. 

Pre- and probiotic feed additives can include the use of *Lactobacillus*. Administering certain *Lactobacillus* species to healthy mice has modulated the expression of specific GABA receptors involved in anxiety behaviours [6]. This increase in abundance of specific species such as *L. rhamnosus* has been shown to decrease the production of stress-induced corticosterone and reduce anxiety and depressive behaviour in mice [6,100], while the administration of *L. paracasei* can reduce the presence of *Clostridium* and Enterobacteriales in pigs, both of which are related to depression and gut dysbiosis [101]. Administration of commensal bacteria such as *Bifidobacterium infantis* can influence the HPA axis by reducing plasma adrenocorticotropic hormone and cortisol levels under laboratory conditions [11]. 

Another potentially beneficial dietary supplement is spray-dried porcine plasma (SDPP), which has been shown to alleviate neuroinflammation and improve memory, aiding in preventing the cognitive decline seen in Alzheimer’s syndrome [102]. SDPP contains many functional components, such as immunoglobulins, growth factors, transferrin, and many other peptides, which can be beneficial to the production of anti-inflammatory gut bacteria in mice [102]. This supplementation promotes abundance of the Lactobacillaceae family and *Acetobacterium*, which has been shown to produce acetyl-CoA, improve cognitive function during ageing, and act as a key precursor of butyrate [102]. SDPP supplementation simultaneously reduced the pro-inflammatory effects of pathogenic bacteria, such as *Johnsonella* and *Erysipelothrix*, preventing the marked effects of ageing seen in Alzheimer’s patients [102]. This remarkable additive may have future use in the treatment or prevention of other mental disorders by eliciting similar effects on the brain and gut microbiota. While there is great potential for additives such as these to play a permanent role in the health and performance of humans and animals alike, further evidence-based studies (such as double-blind placebo-controlled clinical trials) are required before they can be recommended in any treatment regime. Calorie restriction in the diet is another potential beneficial strategy to decrease anxiety and depression [98]. 

Research exploring the connections between animal behaviour and gut microbiota is limited, as studies have, for the most part, used partial 16S rRNA amplicon sequencing and involved small sample sizes. Furthermore, as this is an emerging field of medical research, there are many unknowns regarding the composition, function, and effect of the microbiota. These knowledge gaps are even more extensive in the understudied microbiomes of animals other than laboratory rodents. Additionally, in human and animal-focused research, gut microbiota profiling is often generalised for the entire gastrointestinal tract using a single faecal sample, which may not be representative of the different gastrointestinal regions and does not account for the microbial diversity within individual sections of the tract, which have their own unique microbiota in terms of individual species and relative abundance [103,104,105,106,107]. Sex and age also can strongly influence the association between gut microbiome and behaviour and need to be considered in future research [108].

It should be noted that the current study has only focused on the bacterial component of the gut microbiota. However, the gut microbiota is a complex ecosystem harbouring bacteria, fungi, and, more importantly, viruses [109,110,111]. The gut contains plant-derived viruses, giant viruses, and bacteriophages that interact with bacteria [109]. In particular, phages may have a large impact on the gut microbiota composition as they are obligate intracellular viruses that infect bacteria, causing lysis, as well as being vehicles for horizontal gene transfer within and between species. Additionally, phages may contribute to the host immune response [112,113], which can influence behavioural issues and other host diseases. Phages are capable of transcellular transport across the gut epithelial layer and can enter the blood stream in enormous quantities [114,115,116]. In humans, it has been estimated that each day, around 10^13^ phages cross the wall of the gut and enter the bloodstream [114,115]. Phages are detected in all main organs, including the lung, liver, kidney, spleen, and brain [114,115]. Identification of phages in the brain highlights their capacity to pass the highly controlled blood-brain barrier, affect brain immunity, and possibly regulate behavioural issues and other neurological diseases. Detection of phages by pattern recognition receptors can trigger the innate immune response [117]. Phages can also stimulate the adaptive immune response [118]. Bacteriophages can be considered novel candidates for significant roles in gut dysbiosis and behavioural issues. 

## 5. Conclusions

This systematic review demonstrates that an association exists between gut microbiota diversity and abnormal behavioural phenotypes in production (porcine), performance (equine), and companion (canine) animals. Although no significant differences in alpha diversity in the microbiota were found, differences in beta diversity were observed between affected animals and healthy controls. As seen in humans with mental disorders, animals exhibiting abnormal behaviour often have microbiota population shifts within the phylum Firmicutes, particularly in the families Clostridiaceae, Lachinospiraceae, Oscillospiraceae, and Lactibacillaceae. Within these families, there is often an enrichment of pro-inflammatory and lactic acid-producing bacteria and a reduction in butyrate-producing bacteria in abnormal behavioural phenotypes. To date, however, we are unable to distinguish between cause and effect with regard to microbiota population shifts and abnormal behaviour. The findings indicate that either: (1) abnormal behaviour phenotypes result in physiological changes that alter the gastrointestinal environment and, in turn, influence dysbiosis; (2) specific microbiota may influence the development and severity of abnormal behaviour phenotypes; or (3) animals with abnormal behaviour phenotypes are predisposed to a confounder that also impacts the microbiota. These provide testable hypotheses for future research to establish causal relationships between gut microbiota and abnormal behaviour and offer promising potential for the development of novel therapeutic and/or preventative interventions aimed at restoring a healthy gut-brain-immune axis to mitigate behavioural issues and improve health, performance, and production in animals.

## Figures and Tables

**Table 1:** Microbiota population shifts correlated with mental health disorders in humans.

Behavioural Condition	Study (Reference)	Relative Abundance Increase in Microbiota Composition	Relative Abundance Decrease in Microbiota Composition
**Human**			
Depression	McGuinness et al., 2022 [27]	*Lactobacillus* *Eggerthella* *Enterococcus* *Streptococcus* *Flavonifractor* *Escherichia* *-* *Shigella* *Alistipes* *Parabacteroides* *Veillonella*	*Coprococcus* *Faecalibacterium* *Prevotella* *Ruminococcus*
	Halversion and Alagiakrishnan, 2020 [26]	*Blautia* *Oscillibacter* *Clostridium* *Streptococcus* *Alistipes* *Bacteriodes* *Bifidobacterium* Enterobacteriaceae (family)	*Faecalibacterium* *Lactobacillus* *Prevotella* *Bacteroides* *Bifidobacterium*
	Simpson et al., 2021 [53]	*Lactobacillus* *Oscillibacter* *Blautia* *Streptococcus* *Holdemania* Erysipelotrichaceae (family) *Eggerthella* *Olsenella* *Desulfovibrio*	*Coprococcus* *Faecalibacterium* *Sutterella*
	Alli et al., 2022 [55]	*Streptococcus* *Eggerthella* Bifidobacteriaceae (family)	*Coprococcus* *Faecalibacterium* Sutterellaceae (family)
	Nikolova et al., 2021 [56]	*Eggerthella*	*Faecalibacterium* *Coprococcus*
	Barandouzi et al., 2020 [57]	*Oscillibacter* *Blautia* *Holdemania* *Clostridium* *Anaerostipes* *Streptococcus* *Veillonella* Erysipelotrichaceae (family) *Eubacterium* *Parabacteroides* *Paraprevotella* *Eggerthella* *Olsenella* *Desulfovibrio* *Parasutterella*	*Coprococcus* *Lactobacillus* *Clostridium* *Dialister* *Escherichia-Shigella* *Sutterella*
Anxiety	McGuinness et al., 2022 [27]	*Lactobacillus*	*Coprococcus*
	Halversion and Alagiakrishnan, 2020 [26]	*Clostridium* *Ruminococcus* *Escherischia-Shigella*	*Bacteroides*
	Nikolova et al., 2021 [56]	*Eggerthella*	*Coprococcus* *Faecalibacterium*
Bipolar disorder	McGuinness et al., 2022 [27]	*Lactobacillus* *Enterococcus* *Streptococcus* *Bifidobacterium* *Oscillibacter* *Megasphaera* *Eggerthella* *Flavonifractor*	*Coprococcus* *Roseburia* *Faecalibacterium* *Ruminococcus*
	Nikolova et al., 2021 [56]	*Eggerthella*	*Coprococcus* *Faecalibacterium*
	Halverson and Alagiakrishnan, 2020 [26]		*Faecalibacterium*
Schizophrenia	McGuinness et al., 2022 [27]	*Lactobacillus* *Escherischia-Shigella* *Veillonella* *Eggerthella* *Megasphaera* *Prevotella*	*Coprococcus* *Roseburia* *Bacteroides* *Haemophilus * *Streptococcus*
	Nikolova et al., 2021 [56]	*Eggerthella*	*Coprococcus* *Faecalibacterium*
	Halverson and Alagiakrishnan, 2020 [26]	*Escherischia-Shigella* *Succinivibrio*	*Coprococcus*, *Roseburia* *Faecalibacterium* Ruminococcaceae (family)

**Table 2:** Microbiota population shifts correlated with specific behavioural disorders in three animal species (porcine, equine, and canine).

Behavioural Condition	Study	Increased	Decreased
Porcine			
Tail biter (Aggressor)	Rabhi et al., 2020 [66]	*Coprococcus* *Clostridium*	*Lactobacillus* *Butyricicoccus* *Pseudobutyrivibrio* *Roseburia* *Anaeroplasma*
	Verbeek et al., 2021 [67]	Firmicutes (phylum) Clostridiales (order) Lachnospiraceae (family) Ruminococcaceae (family) *Ruminiclostridium* Family_XIII_AD3011	
Tail-bitten (Victim)	Rabhi et al., 2020 [66]	*Sphaerochaeta* *Blautia* *Alistipes*	*Lactobacillus* *Intestinimonas*
	Verbeek et al., 2021 [67]	*Alloprevotella* *Butyrivibrio* Lachnospiraceae (family)	*Prevotella* *Ralstonia*
Fearful behaviour/lack of exploratory behaviour	Choudhury et al., 2022 [4]	*Prevotella/Prevotellaceae* *Eubacterium* *Coprostanoligenes* *Coprococcus*	
Equine			
Hypervigilance and alertness	Mach et al., 2020 [68]	*Dehalobacterium* *Denitrobacterium*	
	Destrez et al., 2019 [69]	Succinivibrionaceae (family)	
	Bulmer et al., 2019 [70]	*Streptococcus*	Ruminococcaceae (family)
Unresponsive and withdrawn	Mach et al., 2020 [68]	*Anaerorhabdus* *Diplorickettsia* *Novosphyngobium*	
	Mach et al., 2021 [49]	Lachnospiraceae (family) Clostridiales (family)	
Aggression	Mach et al., 2020 [68]	*Streptococcus* *Butyrivibrio*	*Anaeroplasma*
Oral stereotypies	Mach et al., 2020 [68]	*Roseburia* *Desulfurispora* *Pseudobacteroides* *Acinetobacter* *Helicobacter* *Ruminobacter* *Marinilabiliaceae (family)*	
Locomotion stereotypies	Mach et al., 2020 [68]	*Streptomyces*	
Canine			
Phobia	Craddock et al., 2022 [71]	*Lactobacillus*	
	Mondo et al., 2020 [72]	*Lactobacillus* Rikenellaceae (family)	
Aggression	Craddock et al., 2022 [71]	*Ruminococcus*	
	Kirchoff et al., 2019 [61]	*Lactobacillus*	
	Mondo et al., 2020 [72]	*Catenibacterium* *Megamonas* *Eubacterium*	*Oscillospira* *Peptostreptococcus* *Bacteroides* *Sutterella*

## Data Availability

All data are extracted from the literature in PubMed and the references are cited in the manuscript.

## References

[1] Casertano M., Fogliano V., Ercolini D. (2022). Psychobiotics, gut microbiota and fermented foods can help preserving mental health. Food Res. Int..

[2] Berding K., Cryan J.F. (2022). Microbiota-targeted interventions for mental health. Curr. Opin. Psychiatry.

[3] Liu T., Feenstra K.A., Heringa J., Huang Z. (2020). Influence of gut microbiota on mental health via neurotransmitters: A review. J. Artif. Intell. Med. Sci..

[4] Choudhury R., Middelkoop A., Bolhuis J., Kleerebezem M. (2022). Exploring the association between microbiota and behaviour in suckling piglets. Sci. Rep..

[5] Patil Y., Gooneratne R., Ju X.-H. (2020). Interactions between host and gut microbiota in domestic pigs: A review. Gut Microbes.

[6] Bravo J.A., Forsythe P., Chew M.V., Escaravage E., Savignac H.M., Dinan T.G., Bienenstock J., Cryan J.F. (2011). Ingestion of *Lactobacillus* strain regulates emotional behavior and central GABA receptor expression in a mouse via the vagus nerve. Proc. Natl. Acad. Sci. USA.

[7] Browne H.P., Shao Y., Lawley T.D. (2022). Mother–infant transmission of human microbiota. Curr. Opin. Microbiol..

[8] Yang L., Sakandar H.A., Sun Z., Zhang H. (2021). Recent advances of intestinal microbiota transmission from mother to infant. J. Funct. Foods.

[9] Fields R.D. (2008). White matter in learning, cognition and psychiatric disorders. Trends Neurosci..

[10] Yip S., Dehcheshmeh M.M., McLelland D.J., Boardman W.S., Saputra S., Ebrahimie E., Weyrich L.S., Bird P.S., Trott D.J. (2021). *Porphyromonas* spp., *Fusobacterium* spp., and *Bacteroides* spp. dominate microbiota in the course of macropod progressive periodontal disease. Sci. Rep..

[11] Chen L.L., Abbaspour A., Mkoma G.F., Bulik C.M., Rück C., Djurfeldt D. (2021). Gut microbiota in psychiatric disorders: A systematic review. Psychosom. Med..

[12] Chen S., Luo S., Yan C. (2021). Gut Microbiota Implications for Health and Welfare in Farm Animals: A Review. Animals.

[13] Fan L., Liu B., Han Z., Ren W. (2021). Insights into host-microbe interaction: What can we do for the swine industry?. Anim. Nutr..

[14] Lee M., Jeong S., Seo J., Seo S. (2019). Changes in the ruminal fermentation and bacterial community structure by a sudden change to a high-concentrate diet in Korean domestic ruminants. Asian-Australas. J. Anim. Sci..

[15] Henry M., Jansen H., Amezcua M.d.R., O’Sullivan T.L., Niel L., Shoveller A.K., Friendship R.M. (2021). Tail-biting in pigs: A scoping review. Animals.

[16] Rhee S.H., Pothoulakis C., Mayer E.A. (2009). Principles and clinical implications of the brain–gut–enteric microbiota axis. Nat. Rev. Gastroenterol. Hepatol..

[17] Clarke G., Stilling R.M., Kennedy P.J., Stanton C., Cryan J.F., Dinan T.G. (2014). Minireview: Gut microbiota: The neglected endocrine organ. Mol. Endocrinol..

[18] Moher D., Liberati A., Tetzlaff J., Altman D.G., the PRISMA Group (2009). Preferred reporting items for systematic reviews and meta-analyses: The PRISMA statement. Ann. Intern. Med..

[19] Backhed F., Ley R.E., Sonnenburg J.L., Peterson D.A., Gordon J.I. (2005). Host-bacterial mutualism in the human intestine. Science.

[20] Neish A.S. (2009). Microbes in gastrointestinal health and disease. Gastroenterology.

[21] Misiak B., Łoniewski I., Marlicz W., Frydecka D., Szulc A., Rudzki L., Samochowiec J. (2020). The HPA axis dysregulation in severe mental illness: Can we shift the blame to gut microbiota?. Prog. Neuro-Psychopharmacol. Biol. Psychiatry.

[22] Makris A.P., Karianaki M., Tsamis K.I., Paschou S.A. (2021). The role of the gut-brain axis in depression: Endocrine, neural, and immune pathways. Hormones.

[23] Silva Y.P., Bernardi A., Frozza R.L. (2020). The role of short-chain fatty acids from gut microbiota in gut-brain communication. Front. Endocrinol..

[24] Sylvia K.E., Demas G.E. (2018). A gut feeling: Microbiome-brain-immune interactions modulate social and affective behaviors. Horm. Behav..

[25] Dash S., Syed Y.A., Khan M.R. (2022). Understanding the role of the gut microbiome in brain development and its association with neurodevelopmental psychiatric disorders. Front. Cell Dev. Biol..

[26] Halverson T., Alagiakrishnan K. (2020). Gut microbes in neurocognitive and mental health disorders. Ann. Med..

[27] McGuinness A., Davis J., Dawson S., Loughman A., Collier F., O’Hely M., Simpson C., Green J., Marx W., Hair C. (2022). A systematic review of gut microbiota composition in observational studies of major depressive disorder, bipolar disorder and schizophrenia. Mol. Psychiatry.

[28] Chu C., Murdock M.H., Jing D., Won T.H., Chung H., Kressel A.M., Tsaava T., Addorisio M.E., Putzel G.G., Zhou L. (2019). The microbiota regulate neuronal function and fear extinction learning. Nature.

[29] Hong S., Beja-Glasser V.F., Nfonoyim B.M., Frouin A., Li S., Ramakrishnan S., Merry K.M., Shi Q., Rosenthal A., Barres B.A. (2016). Complement and microglia mediate early synapse loss in Alzheimer mouse models. Science.

[30] Wilton D.K., Dissing-Olesen L., Stevens B. (2019). Neuron-glia signaling in synapse elimination. Annu. Rev. Neurosci..

[31] Van Erp J.B.F. (2022). Gastrointestinal tract-based implicit measures for cognition, emotion and behavior. Front. Comput. Sci..

[32] Luca M., Chattipakorn S.C., Sriwichaiin S., Luca A. (2020). Cognitive-behavioural correlates of dysbiosis: A review. Int. J. Mol. Sci..

[33] Dalile B., Van Oudenhove L., Vervliet B., Verbeke K. (2019). The role of short-chain fatty acids in microbiota–gut–brain communication. Nat. Rev. Gastroenterol. Hepatol..

[34] Bajaj M.K. (2020). Neurotransmitter and Behaviour. Examining Biological Foundations of Human Behavior.

[35] Wang F., Yang J., Pan F., Ho R.C., Huang J.H. (2020). Editorial: Neurotransmitters and Emotions. Front. Psychol..

[36] Kaur H., Bose C., Mande S.S. (2019). Tryptophan metabolism by gut microbiome and gut-brain-axis: An in silico analysis. Front. Neurosci..

[37] Yano J.M., Yu K., Donaldson G.P., Shastri G.G., Ann P., Ma L., Nagler C.R., Ismagilov R.F., Mazmanian S.K., Hsiao E.Y. (2015). Indigenous bacteria from the gut microbiota regulate host serotonin biosynthesis. Cell.

[38] Chen Y., Xu J., Chen Y. (2021). Regulation of neurotransmitters by the gut microbiota and effects on cognition in neurological disorders. Nutrients.

[39] Morris G., Berk M., Carvalho A., Caso J.R., Sanz Y., Walder K., Maes M. (2017). The role of the microbial metabolites including tryptophan catabolites and short chain fatty acids in the pathophysiology of immune-inflammatory and neuroimmune disease. Mol. Neurobiol..

[40] Kuwahara A., Matsuda K., Kuwahara Y., Asano S., Inui T., Marunaka Y. (2020). Microbiota-gut-brain axis: Enteroendocrine cells and the enteric nervous system form an interface between the microbiota and the central nervous system. Biomed. Res..

[41] Hansen M.B., Witte A.B. (2008). The role of serotonin in intestinal luminal sensing and secretion. Acta Physiol..

[42] Caspani G., Swann J. (2019). Small talk: Microbial metabolites involved in the signaling from microbiota to brain. Curr. Opin. Pharmacol..

[43] Rho J.M., Storey T.W. (2001). Molecular ontogeny of major neurotransmitter receptor systems in the mammalian central nervous system: Norepinephrine, dopamine, serotonin, acetylcholine, and glycine. J. Child Neurol..

[44] Mittal R., Debs L.H., Patel A.P., Nguyen D., Patel K., O’Connor G., Grati M.h., Mittal J., Yan D., Eshraghi A.A. (2017). Neurotransmitters: The critical modulators regulating gut–brain axis. J. Cell. Physiol..

[45] Frost G., Sleeth M.L., Sahuri-Arisoylu M., Lizarbe B., Cerdan S., Brody L., Anastasovska J., Ghourab S., Hankir M., Zhang S. (2014). The short-chain fatty acid acetate reduces appetite via a central homeostatic mechanism. Nat. Commun..

[46] Tynes V.V., Landsberg G.M. (2021). Nutritional management of behavior and brain disorders in dogs and cats. Vet. Clin. Small Anim. Pract..

[47] Mondo E., Marliani G., Accorsi P.A., Cocchi M., Di Leone A. (2019). Role of gut microbiota in dog and cat’s health and diseases. Open Vet. J..

[48] Packard A.E., Egan A.E., Ulrich-Lai Y.M. (2016). HPA axis-Interaction with Behavioral Systems. Compr. Physiol..

[49] Mach N., Lansade L., Bars-Cortina D., Dhorne-Pollet S., Foury A., Moisan M.-P., Ruet A. (2021). Gut microbiota resilience in horse athletes following holidays out to pasture. Sci. Rep..

[50] Luo Y., Zeng B., Zeng L., Du X., Li B., Huo R., Liu L., Wang H., Dong M., Pan J. (2018). Gut microbiota regulates mouse behaviors through glucocorticoid receptor pathway genes in the hippocampus. Transl. Psychiatry.

[51] Dupont H.L., Jiang Z.-D., Dupont A.W., Utay N.S. (2020). The intestinal microbiome in human health and disease. Trans. Am. Clin. Climatol. Assoc..

[52] Diz-Chaves Y., Astiz M., Bellini M.J., Garcia-Segura L.M. (2013). Prenatal stress increases the expression of proinflammatory cytokines and exacerbates the inflammatory response to LPS in the hippocampal formation of adult male mice. Brain Behav. Immun..

[53] Simpson C.A., Diaz-Arteche C., Eliby D., Schwartz O.S., Simmons J.G., Cowan C.S. (2021). The gut microbiota in anxiety and depression–A systematic review. Clin. Psychol. Rev..

[54] Simpson C.A., Mu A., Haslam N., Schwartz O.S., Simmons J.G. (2020). Feeling down? A systematic review of the gut microbiota in anxiety/depression and irritable bowel syndrome. J. Affect. Disord..

[55] Alli S.R., Gorbovskaya I., Liu J.C., Kolla N.J., Brown L., Müller D.J. (2022). The Gut Microbiome in Depression and Potential Benefit of Prebiotics, Probiotics and Synbiotics: A Systematic Review of Clinical Trials and Observational Studies. Int. J. Mol. Sci..

[56] Nikolova V.L., Hall M.R., Hall L.J., Cleare A.J., Stone J.M., Young A.H. (2021). Perturbations in gut microbiota composition in psychiatric disorders: A review and meta-analysis. JAMA Psychiatry.

[57] Barandouzi Z.A., Starkweather A.R., Henderson W.A., Gyamfi A., Cong X.S. (2020). Altered composition of gut microbiota in depression: A systematic review. Front. Psychiatry.

[58] Walters K.E., Martiny J.B. (2020). Alpha-, beta-, and gamma-diversity of bacteria varies across habitats. PLoS ONE.

[59] Bharwani A., Mian M.F., Surette M.G., Bienenstock J., Forsythe P. (2017). Oral treatment with Lactobacillus rhamnosus attenuates behavioural deficits and immune changes in chronic social stress. BMC Med..

[60] Val-Laillet D., Guérin S., Coquery N., Nogret I., Formal M., Romé V., Le Normand L., Meurice P., Randuineau G., Guilloteau P. (2018). Oral sodium butyrate impacts brain metabolism and hippocampal neurogenesis, with limited effects on gut anatomy and function in pigs. FASEB J..

[61] Kirchoff N.S., Udell M.A., Sharpton T.J. (2019). The gut microbiome correlates with conspecific aggression in a small population of rescued dogs (*Canis familiaris*). PeerJ.

[62] Garner J.P. (2005). Stereotypies and other abnormal repetitive behaviors: Potential impact on validity, reliability, and replicability of scientific outcomes. ILAR J..

[63] Hanis F., Chung E.L.T., Kamalludin M.H., Idrus Z. (2020). Discovering the relationship between dietary nutrients and cortisol and ghrelin hormones in horses exhibiting oral stereotypic behaviors: A review. J. Vet. Behav..

[64] David L.A., Maurice C.F., Carmody R.N., Gootenberg D.B., Button J.E., Wolfe B.E., Ling A.V., Devlin A.S., Varma Y., Fischbach M.A. (2014). Diet rapidly and reproducibly alters the human gut microbiome. Nature.

[65] Sonnenburg J.L., Bäckhed F. (2016). Diet–microbiota interactions as moderators of human metabolism. Nature.

[66] Rabhi N., Thibodeau A., Côté J.-C., Devillers N., Laplante B., Fravalo P., Larivière-Gauthier G., Thériault W.P., Faucitano L., Beauchamp G. (2020). Association between tail-biting and intestinal microbiota composition in pigs. Front. Vet. Sci..

[67] Verbeek E., Dicksved J., Keeling L. (2021). Supplementation of Lactobacillus early in life alters attention bias to threat in piglets. Sci. Rep..

[68] Mach N., Ruet A., Clark A., Bars-Cortina D., Ramayo-Caldas Y., Crisci E., Pennarun S., Dhorne-Pollet S., Foury A., Moisan M.-P. (2020). Priming for welfare: Gut microbiota is associated with equitation conditions and behavior in horse athletes. Sci. Rep..

[69] Destrez A., Grimm P., Julliand V. (2019). Dietary-induced modulation of the hindgut microbiota is related to behavioral responses during stressful events in horses. Physiol. Behav..

[70] Bulmer L.S., Murray J.-A., Burns N.M., Garber A., Wemelsfelder F., McEwan N.R., Hastie P.M. (2019). High-starch diets alter equine faecal microbiota and increase behavioural reactivity. Sci. Rep..

[71] Craddock H.A., Godneva A., Rothschild D., Motro Y., Grinstein D., Lotem-Michaeli Y., Narkiss T., Segal E., Moran-Gilad J. (2022). Phenotypic correlates of the working dog microbiome. Npj Biofilms Microbiomes.

[72] Mondo E., Barone M., Soverini M., D’amico F., Cocchi M., Petrulli C., Mattioli M., Marliani G., Candela M., Accorsi P. (2020). Gut microbiome structure and adrenocortical activity in dogs with aggressive and phobic behavioral disorders. Heliyon.

[73] Verbeek E., Keeling L., Landberg R., Lindberg J.E., Dicksved J. (2021). The gut microbiota and microbial metabolites are associated with tail biting in pigs. Sci. Rep..

[74] Konstantinov S.R., Smidt H., de Vos W.M., Bruijns S.C., Singh S.K., Valence F., Molle D., Lortal S., Altermann E., Klaenhammer T.R. (2008). S layer protein A of Lactobacillus acidophilus NCFM regulates immature dendritic cell and T cell functions. Proc. Natl. Acad. Sci. USA.

[75] Lebeer S., Vanderleyden J., De Keersmaecker S.C. (2010). Host interactions of probiotic bacterial surface molecules: Comparison with commensals and pathogens. Nat. Rev. Microbiol..

[76] Bermudez-Brito M., Plaza-Díaz J., Muñoz-Quezada S., Gómez-Llorente C., Gil A. (2012). Probiotic mechanisms of action. Ann. Nutr. Metab..

[77] Hamamah S., Aghazarian A., Nazaryan A., Hajnal A., Covasa M. (2022). Role of microbiota-gut-brain axis in regulating dopaminergic signaling. Biomedicines.

[78] Ephraim E., Brockman J.A., Jewell D.E. (2022). A Diet Supplemented with Polyphenols, Prebiotics and Omega-3 Fatty Acids Modulates the Intestinal Microbiota and Improves the Profile of Metabolites Linked with Anxiety in Dogs. Biology.

[79] McGowan R.T., Barnett H.R., Czarnecki-Maulden G., Si X., Perez-Camargo G., Martin F. Tapping into those ‘gut feelings’: Impact of BL999 (Bifidobacterium longum) on anxiety in dogs. Proceedings of the Veterinary Behavior Symposium Proceedings.

[80] Coccaro E.F., Fanning J.R., Phan K.L., Lee R. (2015). Serotonin and impulsive aggression. CNS Spectr..

[81] Reisner I.R., Mann J.J., Stanley M., Huang Y.-y., Houpt K.A. (1996). Comparison of cerebrospinal fluid monoamine metabolite levels in dominant-aggressive and non-aggressive dogs. Brain Res..

[82] Hsiao E.Y., McBride S.W., Hsien S., Sharon G., Hyde E.R., McCue T., Codelli J.A., Chow J., Reisman S.E., Petrosino J.F. (2013). Microbiota modulate behavioral and physiological abnormalities associated with neurodevelopmental disorders. Cell.

[83] Nicol C. (1999). Understanding equine stereotypies. Equine Vet. J..

[84] Sarrafchi A., Blokhuis H.J. (2013). Equine stereotypic behaviors: Causation, occurrence, and prevention. J. Vet. Behav..

[85] Johnson K., Tyrrell J., Rowe J., Pethick D. (1998). Behavioural changes in stabled horses given nontherapeutic levels of virginiamycin. Equine Vet. J..

[86] Hanis F., Chung E.L.T., Kamalludin M.H., Idrus Z. (2021). Do nutrient composition of feedstuffs affect the proportion of oral stereotypies and redirected behaviors among horse working groups?. J. Vet. Behav..

[87] Garber A., Hastie P., Murray J.-A. (2020). Factors influencing equine gut microbiota: Current knowledge. J. Equine Vet. Sci..

[88] Destrez A., Grimm P., Cézilly F., Julliand V. (2015). Changes of the hindgut microbiota due to high-starch diet can be associated with behavioral stress response in horses. Physiol. Behav..

[89] Milinovich G., Trott D., Burrell P., Van Eps A., Thoefner M., Blackall L., Al Jassim R., Morton J., Pollitt C. (2006). Changes in equine hindgut bacterial populations during oligofructose-induced laminitis. Environ. Microbiol..

[90] Morrison D.J., Preston T. (2016). Formation of short chain fatty acids by the gut microbiota and their impact on human metabolism. Gut Microbes.

[91] Jiang H., Ling Z., Zhang Y., Mao H., Ma Z., Yin Y., Wang W., Tang W., Tan Z., Shi J. (2015). Altered fecal microbiota composition in patients with major depressive disorder. Brain Behav. Immun..

[92] Zheng P., Zeng B., Zhou C., Liu M., Fang Z., Xu X., Zeng L., Chen J., Fan S., Du X. (2016). Gut microbiome remodeling induces depressive-like behaviors through a pathway mediated by the host’s metabolism. Mol. Psychiatry.

[93] Valles-Colomer M., Falony G., Darzi Y., Tigchelaar E.F., Wang J., Tito R.Y., Schiweck C., Kurilshikov A., Joossens M., Wijmenga C. (2019). The neuroactive potential of the human gut microbiota in quality of life and depression. Nat. Microbiol..

[94] Pootakham W., Mhuantong W., Yoocha T., Putchim L., Sonthirod C., Naktang C., Thongtham N., Tangphatsornruang S. (2017). High resolution profiling of coral-associated bacterial communities using full-length 16S rRNA sequence data from PacBio SMRT sequencing system. Sci. Rep..

[95] Franzén O., Hu J., Bao X., Itzkowitz S.H., Peter I., Bashir A. (2015). Improved OTU-picking using long-read 16S rRNA gene amplicon sequencing and generic hierarchical clustering. Microbiome.

[96] Zeineldin M., Aldridge B., Lowe J. (2018). Dysbiosis of the fecal microbiota in feedlot cattle with hemorrhagic diarrhea. Microb. Pathog..

[97] Menneson S., Ménicot S., Malbert C.-H., Meurice P., Serrand Y., Noirot V., Etienne P., Coquery N., Val-Laillet D. (2020). Neuromodulatory and possible anxiolytic-like effects of a spice functional food ingredient in a pig model of psychosocial chronic stress. J. Funct. Foods.

[98] Govic A., Nasser H., Levay E.A., Zelko M., Ebrahimie E., Dehcheshmeh M.M., Kent S., Penman J., Hazi A. (2022). Long-Term Calorie Restriction Alters Anxiety-like Behaviour and the Brain and Adrenal Gland Transcriptomes of the Ageing Male Rat. Nutrients.

[99] Price J.L., Drevets W.C. (2010). Neurocircuitry of mood disorders. Neuropsychopharmacology.

[100] Dinan T.G., Cryan J.F. (2012). Regulation of the stress response by the gut microbiota: Implications for psychoneuroendocrinology. Psychoneuroendocrinology.

[101] Valeriano V., Balolong M., Kang D.K. (2017). Probiotic roles of *Lactobacillus* sp. in swine: Insights from gut microbiota. J. Appl. Microbiol..

[102] Rosell-Cardona C., Amat C., Griñán-Ferré C., Polo J., Pallàs M., Pérez-Bosque A., Moretó M., Miró L. (2022). The Neuroprotective Effects of Spray-Dried Porcine Plasma Supplementation Involve the Microbiota−Gut−Brain Axis. Nutrients.

[103] Alfano N., Courtiol A., Vielgrader H., Timms P., Roca A.L., Greenwood A.D. (2015). Variation in koala microbiomes within and between individuals: Effect of body region and captivity status. Sci. Rep..

[104] Jalanka J., Major G., Murray K., Singh G., Nowak A., Kurtz C., Silos-Santiago I., Johnston J.M., de Vos W.M., Spiller R. (2019). The effect of psyllium husk on intestinal microbiota in constipated patients and healthy controls. Int. J. Mol. Sci..

[105] Rinninella E., Raoul P., Cintoni M., Franceschi F., Miggiano G.A.D., Gasbarrini A., Mele M.C. (2019). What is the healthy gut microbiota composition? A changing ecosystem across age, environment, diet, and diseases. Microorganisms.

[106] Durban A., Abellan J.J., Jimenez-Hernandez N., Latorre A., Moya A. (2012). Daily follow-up of bacterial communities in the human gut reveals stable composition and host-specific patterns of interaction. FEMS Microbiol. Ecol..

[107] Carroll I.M., Ringel-Kulka T., Keku T.O., Chang Y.-H., Packey C.D., Sartor R.B., Ringel Y. (2011). Molecular analysis of the luminal-and mucosal-associated intestinal microbiota in diarrhea-predominant irritable bowel syndrome. Am. J. Physiol.-Gastrointest. Liver Physiol..

[108] Chen J.J., Zheng P., Liu Y.Y., Zhong X.G., Wang H.Y., Guo Y.J., Xie P. (2018). Sex differences in gut microbiota in patients with major depressive disorder. Neuropsychiatr. Dis. Treat..

[109] Scarpellini E., Ianiro G., Attili F., Bassanelli C., De Santis A., Gasbarrini A. (2015). The human gut microbiota and virome: Potential therapeutic implications. Dig. Liver Dis..

[110] Satokari R. (2019). Modulation of gut microbiota for health by current and next-generation probiotics. Nutrients.

[111] Ruppé É., Lisboa T., Barbier F. (2018). The gut microbiota of critically ill patients: First steps in an unexplored world. Intensive Care Med..

[112] Scarpellini E., Fagoonee S., Rinninella E., Rasetti C., Aquila I., Larussa T., Ricci P., Luzza F., Abenavoli L. (2020). Gut microbiota and liver interaction through immune system cross-talk: A comprehensive review at the time of the SARS-CoV-2 pandemic. J. Clin. Med..

[113] Gogokhia L., Buhrke K., Bell R., Hoffman B., Brown D.G., Hanke-Gogokhia C., Ajami N.J., Wong M.C., Ghazaryan A., Valentine J.F. (2019). Expansion of bacteriophages is linked to aggravated intestinal inflammation and colitis. Cell Host Microbe.

[114] Geier M.R., Trigg M.E., Merril C.R. (1973). Fate of bacteriophage lambda in non-immune germ-free mice. Nature.

[115] Keller R., Engley F.B. (1958). Fate of bacteriophage particles introduced into mice by various routes. Proc. Soc. Exp. Biol. Med..

[116] Nguyen S., Baker K., Padman B.S., Patwa R., Dunstan R.A., Weston T.A., Schlosser K., Bailey B., Lithgow T., Lazarou M. (2017). Bacteriophage transcytosis provides a mechanism to cross epithelial cell layers. MBio.

[117] Carroll-Portillo A., Lin H.C. (2019). Bacteriophage and the innate immune system: Access and signaling. Microorganisms.

[118] Majewska J., Kaźmierczak Z., Lahutta K., Lecion D., Szymczak A., Miernikiewicz P., Drapała J., Harhala M., Marek-Bukowiec K., Jędruchniewicz N. (2019). Induction of phage-specific antibodies by two therapeutic staphylococcal bacteriophages administered per os. Front. Immunol..

